# 

**Published:** 1860-08

**Authors:** 


OF THE

### Chicago Medical Journal for August, 1860.

ORIGINAL COMMUNICATIONS. PAGE.

ARTICLE 1.—Report of Surgical Cases, treated at the City Hospital during the Summer Term, by Geo. K. Amerman, M. D. Attending Surgeon.......... 441
ARTICLE 2.—Spina Bepida Successfully Treated by Iodine Injections. By D. Brainard, M. D. 450
Chloroform Vapor in Ear-Ache .......... 453
Chlorate of Potash in Mercurial Salivation .......... 453

EDITORIAL.

Proceedings of the Rock River Union Medical Society.......... 454
De Witt County Medical Society.......... 457
Communication from Committee on the Progress of Obstetrics, Ind. State Med. Society..... 459
Notice to Medical Men in Illinois.......... 459
Transactions of the American Medical Association.......... 460

SELECTIONS.

Discussion on Iodism before the French Academy, by M. Velpeau.......... 466
The Laryngoscope.......... 468
Effects of Disease on the Teeth, by Abr. Robertson, D. D. S., M. D.......... 472
Nœuvus Cured by Creasote .......... 476
Swallowing Artificial Teeth.......... 477
The Hospital for Nervous Diseases.......... 477
Subnitrate of Bismuth, in the Treatment of Burns and Scalds. By T. G. Richardson, M. D. 478
Morning Sickness. By Thomas Inman, M. D. .......... 479
Tannin as Antidote to Strychnine. By Professor Kurzac.......... 481
Training of Pugilists.......... 484
Importance of the Functions of the Skin in the Pathology aud Treatment of Tubercular Consumption. By Dr. Toulmin.......... 486
Experimental Pathology—Rational Principles of Therapeutics. By M. Claude Bernard..... 487
Lunatics in the Good Old Times.......... 493
Positions in Parturition. By B. Dowler, M. D. .......... 494
Brooklyn City Hospital. By James M. Minor, M. D.......... 498
The Chicago Zouaves .......... 503
Re-Section of the Entire Radius .......... 504
Death Bed of an Anatomist .......... 504
Ages of Pregnant Women.......... 504
Maternal Influence on Fœtal Deformity.......... 504

